# The changing epidemiological characteristics of severe fever with thrombocytopenia syndrome in China, 2011–2016

**DOI:** 10.1038/s41598-017-08042-6

**Published:** 2017-08-23

**Authors:** Jimin Sun, Liang Lu, Haixia Wu, Jun Yang, Jiangping Ren, Qiyong Liu

**Affiliations:** 1 State Key Laboratory of Infectious Disease Prevention and Control, Collaborative Innovation Center for Diagnosis and Treatment of Infectious Diseases, National Institute for Communicable Disease Control and Prevention, Chinese Center for Disease Control and Prevention, Beiijng, China; 2 Zhejiang Provincial Center for Disease Control and Prevention, Hangzhou, China

## Abstract

Severe fever with thrombocytopenia syndrome (SFTS) is emerging and the number of SFTS cases increased year by year in China. In order to explore the epidemiology trend, we analyzed the changing epidemiological characteristics of SFTS cases in different years and compare characteristics in different provinces. From 2011 to 2016, a total of 5360 laboratory-confirmed SFTS cases were reported and annual case numbers increased year by year. Most SFTS cases occurred in individuals aged between 40 years and 80 years (91.57%), but age distributions of SFTS cases in different years were significantly different and the median ages increased slightly year by year. The numbers of affected counties from 2011 to 2016 increased sharply from 98 to 167. Of note, the seasonal distributions of SFTS cases in different provinces were significantly different (Fisher = 712.157, P = 0.000) and provinces in south regions showed earlier epidemic peak and longer epidemics durations. The median time from illness onset to confirmation of different years was significantly different (χ^2^ = 896.088, P = 0.000) and it peaked in 2014. Furthermore, case fatality rate was associated with province, year, and age of SFTS cases. These results may be helpful for authorities to better preventive strategy and improve interventions against SFTS.

## Introduction

In 2009, a novel virus, named severe fever with thrombocytopenia syndrome virus (SFTSV) was first identified in the rural areas of Hubei Province and Henan Province in central China^1, 2^. SFTSV is classified in the family *Bunyaviridae*, genus *Phlebovirus* and contains three segments of negative or ambisense polarity RNA, designated L, M and S segments. The virus can cause severe fever with thrombocytopenia syndrome (SFTS) and the average case fatality rate of SFTS was about 30% when this disease was firstly discovered. The clinical symptoms of SFTS include fever, fatigue, chill, headache, lymphadenopathy, anorexia, nausea, myalgia, diarrhea, vomiting, abdominal pain, gingival hemorrhage, conjunctival congestion, and so on^3^. Some SFTS patients experience self-limiting clinical course, while some patients decease due to multiple organ failure^4, 5^. Moreover, the clinical symptoms of SFTS are less specific and need to be differentiated from human anaplasmosis and hemorrhagic fever with renal syndrome caused by hantavirus^6, 7^.

After the identification of SFTSV in 2009, the national guideline for prevention and control for SFTS was issued by the Chinese Ministry of Health in 2010 and SFTS patients should be reported by doctors within 24 hours of diagnosis to China Information System for Diseases Control and Prevention. Although confirmed SFTS or SFTS like patients have been reported in South Korea, Japan, United Arab Emirates, and United States, most SFTS cases were reported in China as of 2016^8–11^. In 2011–2012, 2047 cases of SFTS and 129 deaths were reported in over 206 counties of eastern and central China^12^. As the improvement of capacity for SFTSV identification in many areas, more and more SFTS patients were identified in recent years. Here, we analyze the changing epidemiological characteristics of SFTS in different years and different provinces which will provide more detailed information for the prevention of SFTS. Furthermore, factors associated with SFTS fatal outcome are also analyzed to aid the decrease of case fatality rate.

## Materials and Methods

### Case definition

According to the national guideline for prevention and control for SFTS issued by the Chinese Ministry of Health in 2010, SFTS cases were classified as probable or confirmed cases.

An acutely ill person with acute onset of fever (≥38.0 °C) and other symptoms (e.g. gastrointestinal symptoms, bleeding), epidemiological risk factors (being a farmer or being exposed to ticks two weeks before illness onset) and laboratory data consisting of thrombocytopenia and leukocytopenia was defined as a probable case. A probable case with one or more of the following criteria: (1) detection of SFTSV RNA, (2) seroconversion or 4-fold increase in antibody titers between paired serum samples collected at least two weeks apart, and (3) isolation of SFTSV in cell culture^13^ was defined as a confirmed case.

### Data collection

Daily disease surveillance data on SFTS from 2011 to 2016 were obtained from the China Information System for Diseases Control and Prevention. Information of SFTS cases included gender, age, occupation, residential address, date of illness onset, date of confirmation and outcome.

### Data analysis

The age distribution, gender distribution, occupation distribution, seasonal distribution and the intervals from illness onset to confirmation of SFTS cases in different years were summarized using SPSS version 20.0 (Statistical Product and Service Solutions, Chicago, IL, USA). Fisher’s exact test or Wilcoxon Rank Sum W Test were used, as appropriate, to compare the demographic characteristics, seasonal distribution and the intervals from illness onset to confirmation of SFTS cases in different years. All tests were 2-tailed and statistical significance was set at P < 0.05. Seasonal distributions of SFTS cases in different provinces were also analyzed and compared. Geographical distribution of SFTS cases in different years were plotted using ArcGIS 10.1 (ESRI, Redlands, CA, USA). Additionally, single variable analysis and multivariate analysis were conducted to identify factors associated with SFTS fatal outcome using the logistic regression method.

## Results

**Figure 1:**
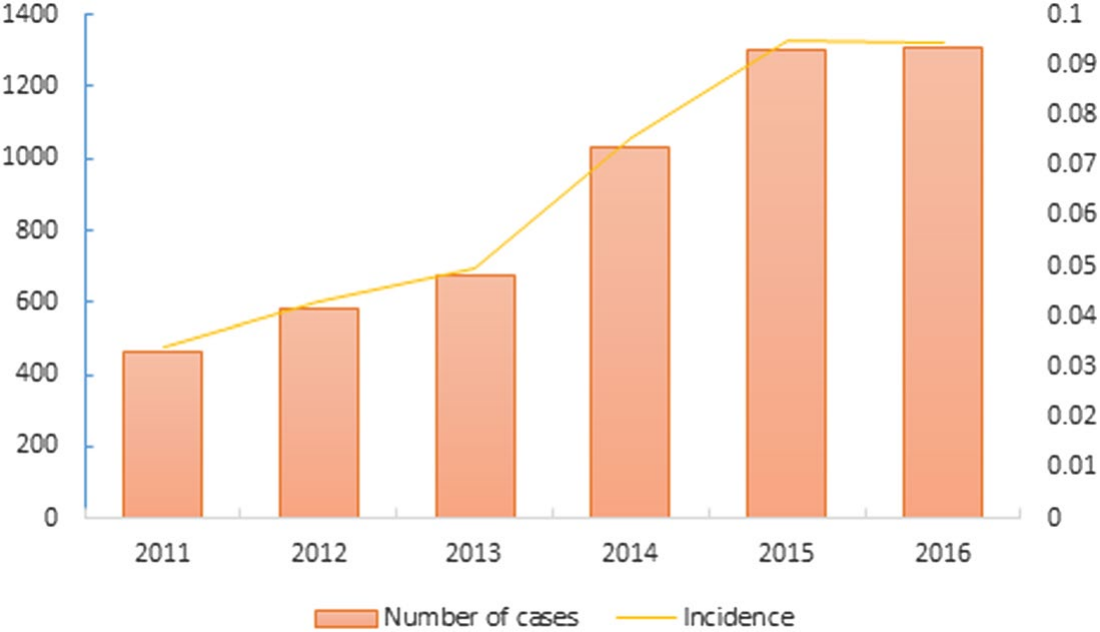
Annual numbers and incidences of SFTS cases in China from 2011 to 2016.

From 2011 to 2016, a total of 5360 laboratory-confirmed cases were reported to China Information System for Diseases Control and Prevention. Annual case numbers increased year by year, with the highest recorded in 2016 (1306 cases, Fig. 1). Of the total SFTS cases, 2509 cases were male and 2851 cases were female. There were more male cases than female cases in 2011, but there were less male cases than female cases from 2012 to 2016.The male-to-female ratio of different years was not significantly different (χ^2^ = 9.526, P = 0.090).

The majority of SFTS cases were farmers (87.91%, 4712/5360) and the occupation distribution of SFTS cases in different years were similar (χ^2^ = 15.552, P = 0.113). Most SFTS cases occurred in individuals aged between 40 years and 80 years (91.57%, Table 1). The median ages of SFTS cases from 2011 to 2016 were 61 years, 60 years, 62 years, 61 years, 63 years, and 63 years, respectively. However, age distributions of SFTS cases in different years were significantly different (Kruskal-Wallis statistic = 23.334, P = 0.000) and the median ages slightly increased from 2011 to 2016.

**Table 1:** Age distribution of SFTS cases in China from 2011 to 2016.

	2011		2012		2013		2014		2015		2016	
	No. of cases	Proportion (%)	No. of cases	Proportion (%)	No. of cases	Proportion (%)	No. of cases	Proportion (%)	No. of cases	Proportion (%)	No. of cases	Proportion (%)
0-	1	0.22	0	0.00	0	0.00	5	0.48	3	0.23	0	0.00
10-	2	0.43	2	0.35	0	0.00	8	0.77	5	0.38	1	0.08
20-	1	0.22	5	0.86	9	1.33	13	1.26	18	1.38	20	1.53
30-	15	3.25	20	3.45	20	2.96	23	2.22	24	1.84	39	2.99
40-	89	19.31	96	16.58	79	11.69	157	15.18	168	12.88	144	11.03
50-	112	24.30	146	25.22	169	25.00	256	24.76	261	20.02	315	24.12
60-	143	31.02	170	29.36	228	33.73	308	29.79	474	36.35	422	32.31
70-	85	18.44	122	21.07	142	21.01	220	21.28	305	23.39	297	22.74
80-	13	2.82	18	3.11	29	4.29	44	4.26	46	3.53	68	5.21
Total	461	100	579	100	676	100	1034	100	1304	100	1306	100

**Figure 2:**
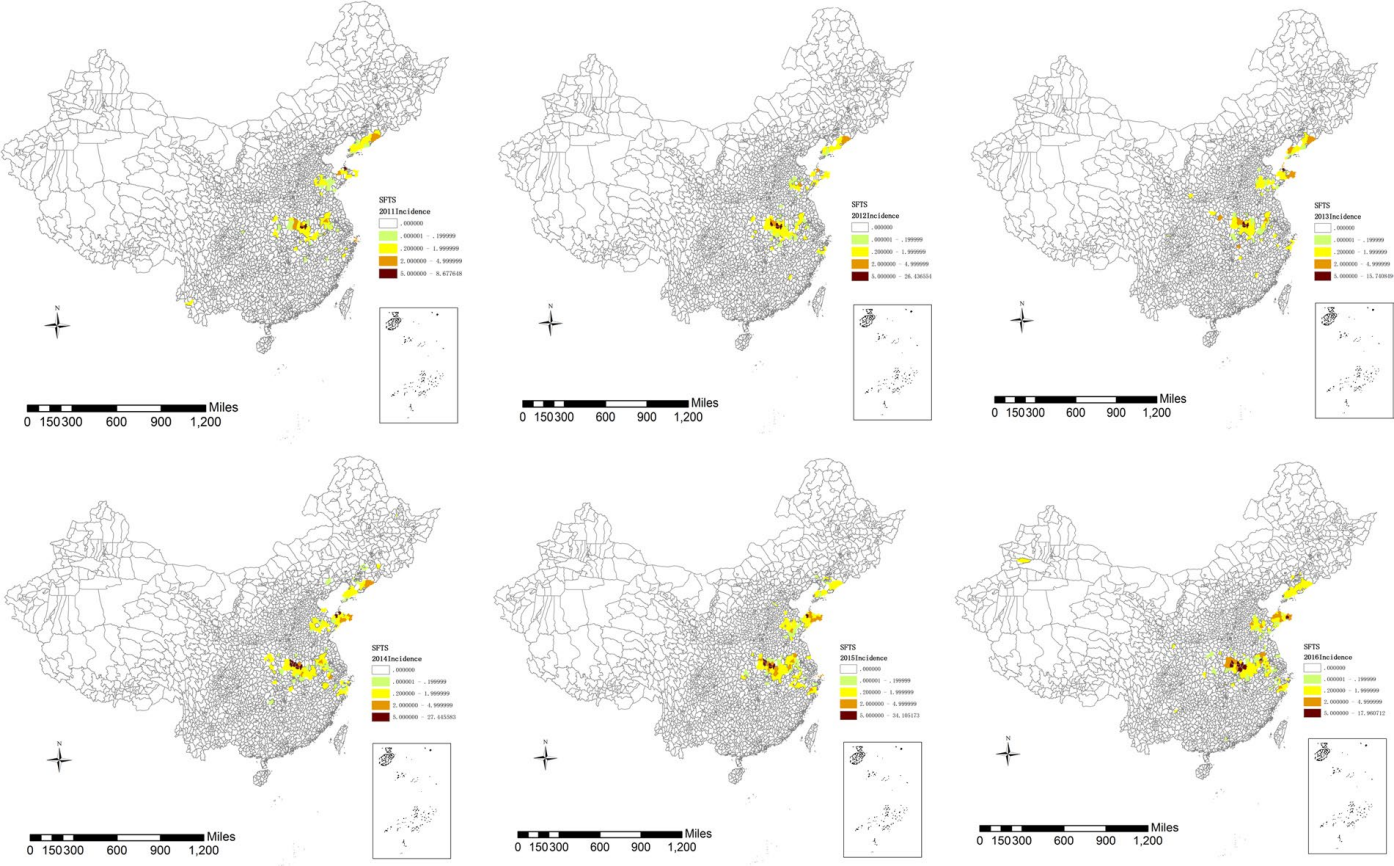
Geographical distribution of SFTS cases in China from 2011 to 2016. These maps were created by ArcGIS software (version 10.1, ESRI Inc.; Redlands, CA, USA). Homepage of ArcGIS software was https://www.esri.com/.

During 2011–2016, 99.53% of SFTS cases were limited to 7 provinces: 2025 cases were reported in Henan Province, 1515 cases in Shandong Province, 663 cases in Hubei Province, 502 cases in Anhui Province, 260 cases in Zhejiang Province, 224 cases in Liaoning Province, and 146 cases in Jiangsu Province. Besides these 7 provinces, other provinces reported only 25 SFTS cases (Table 2). The numbers of affected provinces during 2011 and 2016 were 11, 9, 11, 10, 9, and 11, respectively. However, the numbers of affected counties increased year by year and the numbers were 98, 99, 113, 140, 153, and 167, respectively (Fig. 2).

**Table 2:** Seasonal distribution of SFTS cases in different provinces from 2011 to 2016.

	Liaoning		Shandong		Henan		Hubei		Jiangsu		Anhui		Zhejiang		Other	
	No.	P (%)	No.	P (%)	No.	P (%)	No.	P (%)	No.	P (%)	No.	P (%)	No.	P (%)	No.	P (%)
January	0	0.00	0	0.00	0	0.00	0	0.00	0	0.00	0	0.00	0	0.00	2	8.00
February	0	0.00	0	0.00	0	0.00	0	0.00	0	0.00	0	0.00	0	0.00	1	4.00
March	1	0.45	1	0.07	22	1.09	4	0.61	1	0.68	5	1.00	9	3.46	0	0.00
April	0	0.00	24	1.59	263	12.99	82	12.41	10	6.85	55	10.98	20	7.69	0	0.00
May	10	4.46	271	17.91	616	30.42	168	25.42	33	22.60	110	21.96	57	21.92	1	4.00
June	31	13.84	374	24.72	406	20.05	133	20.12	26	17.81	105	20.96	57	21.92	3	12.00
July	68	30.36	304	20.09	281	13.88	140	21.18	32	21.92	82	16.37	57	21.92	5	20.00
August	67	29.91	276	18.24	137	6.77	58	8.77	20	13.70	64	12.77	26	10.00	4	16.00
September	35	15.63	140	9.25	157	7.75	33	4.99	12	8.22	29	5.79	12	4.62	2	8.00
October	12	5.36	103	6.81	134	6.62	35	5.30	11	7.53	44	8.78	16	6.15	2	8.00
November	0	0.00	20	1.32	9	0.44	8	1.21	1	0.68	7	1.40	5	1.92	4	16.00
December	0	0.00	2	0.13	0	0.00	2	0.30	0	0.00	1	0.20	1	0.38	1	4.00
Total	224	100	1515	100	2025	100	663	100	146	100	502	100	260	100	25	100

*No. = Number of cases, P (%) = Proportion (%).

**Figure 3:**
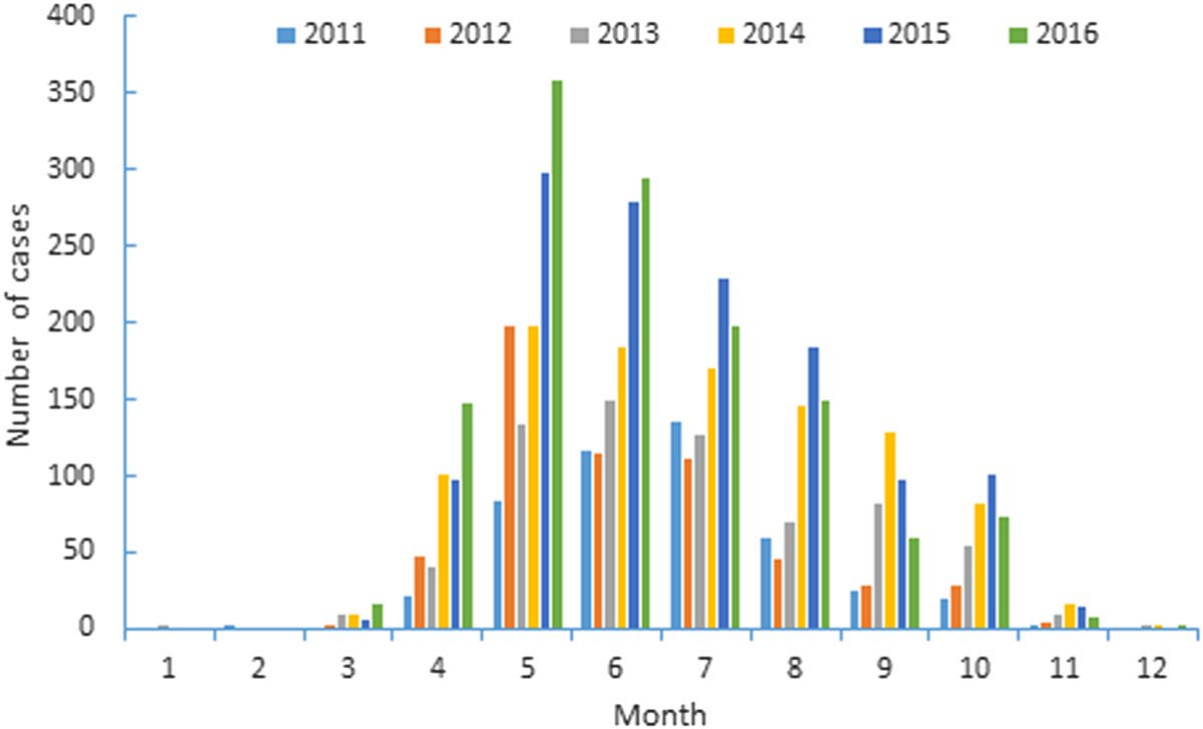
Seasonal distribution of SFTS cases in China from 2011 to 2016.

During 2011–2016, 98.00% (5253/5360) of SFTS cases were reported from April to October with a peak in May, June, and July (Fig. 3). The seasonal distributions of different years were significantly different (Fisher = 276.845, P = 0.000) and the peaks of reported SFTS cases from 2011 to 2016 occurred in July, May, June, May, May, and May, respectively. Of note, the seasonal distributions of SFTS cases in different provinces were significantly different (Fisher = 721.157, P = 0.000). SFTS cases in Liaoning Province showed shorter epidemic periods than other province (Table 2). Moreover, SFTS cases in Zhejiang Province, Hubei Province, Anhui Province, and Henan Province showed earlier peaks than those in Liaoning Province, Shandong Province and Jiangsu Province (April-October vs May-October, Table 2).

The median time from illness onset to confirmation of SFTS cases from 2011 to 2016 were 7 days, 6 days, 6 days, 27 days, 10 days and 12 days, respectively. Notably, the median time from illness onset to confirmation of different years was significantly different (χ^2^ = 896.088, P = 0.000) and it peaked in 2014.

A total of 343 deaths were reported in China from 2011 to 2016 and the average case fatality rate was 6.40%. According to results of single variable logistic regression analysis, there were significant differences in year, province, age and gender. As shown in Table 3, the case fatality rates of SFTS cases in different years were significantly different and it decreased from 2014 to 2016. The case fatality rates of SFTS cases in different provinces were also significantly different and the lowest fatality rate recorded in Henan. The case fatality rate significantly increased with the increase of age of SFTS cases (P = 0.000).

**Table 3:** Single variable analysis on factors associated with SFTS fatal outcome.

	Survivor	Death	Wald	P	OR	95% CI	
Year			26.309	0.000			
2011	415	46	18.532	0.000	2.429	1.622	3.638
2012	536	43	7.321	0.007	1.758	1.168	2.645
2013	628	48	6.523	0.011	1.675	1.127	2.488
2014	953	81	12.185	0.000	1.862	1.314	2.641
2015	1236	68	1.032	0.310	1.206	0.841	1.729
2016	1249	57					
Province			114.367	0.000			
Liaoning	208	16	14.768	0.000	3.132	1.750	5.607
Shandong	1345	170	96.401	0.000	5.101	3.685	7.062
Hubei	618	45	25.926	0.000	2.936	1.940	4.445
Jiangsu	141	5	0.561	0.454	1.430	0.561	3.646
Anhui	478	24	7.695	0.006	2.025	1.230	3.333
Zhejiang	228	32	53.133	0.000	5.660	3.551	9.020
Other	23	2	2.291	0.130	3.102	0.716	13.436
Henan	1976	49					
Age			59.893	0.000	1.561	1.395	1.747
0-	234	0					
40-	715	18					
50-	1188	71					
60-	1628	117					
70-	1252	137					
Gender			3.794	0.051	1.243	0.999	1.547
Female	2686	165					
Male	2331	178					

According to results of multivariable logistic regression analysis, variables in the final equation included year, province and age. Compared with cases in 2016, odds ratio (OR) of cases death in 2011–2015 were 2.836, 2.696, 1.858, 2.271 and 1.426, respectively. Compared with cases in Henan Province, OR of cases death in Liaoning, Shandong, Hubei, Jiangsu, Anhui, Zhejiang and others were 3.212, 5.528, 3.654, 1.653, 2.516, 5.854, and 4.631, respectively. Compared with cases aged 0–39, the case fatality rate increased with the increase of age and OR was1.611 (Table 4).

**Table 4:** Multivariable analysis on factors associated with SFTS fatal outcome.

	Wald	P	OR	95% CI	
Year	38.685	0.000			
2011	24.143	0.000	2.836	1.871	4.298
2012	20.941	0.000	2.696	1.763	4.124
2013	8.943	0.003	1.858	1.238	2.787
2014	20.032	0.000	2.271	1.586	3.252
2015	3.565	0.059	1.426	0.987	2.061
Province	115.110	0.000			
Liaoning	14.922	0.000	3.212	1.777	5.806
Shandong	102.070	0.000	5.528	3.968	7.703
Hubei	35.879	0.000	3.654	2.391	5.584
Jiangsu	1.082	0.298	1.653	0.641	4.257
Anhui	12.740	0.000	2.516	1.516	4.175
Zhejiang	53.359	0.000	5.842	3.638	9.382
Other	4.041	0.044	4.631	1.039	20.637
Age	63.911	0.000	1.611	1.433	1.810

## Discussion

In this study, surveillance data on SFTS from 2011 to 2016 was used to investigate changing epidemiological characteristics of SFTS. We found that the number of SFTS increased year by year. Two factors may contribute the result. First, SFTSV might has been spread to more areas through humans, small mammals, ticks, or birds^14^. Therefore, people in more areas have opportunities to be infected with SFTSV and more SFTS cases occurred. Second, some physicians lacked experience of SFTS diagnosis when SFTSV was identified. Some SFTS cases were misdiagnosed and the reported cases were only the tip of the iceberg. Due to the high fatality rate, more attention have been paid by health departments and some measures have been conducted to improve the capacity of diagnosis and response to SFTS. For example, all municipal center for disease control and prevention established detection methods for SFTSV in Zhejiang Province in 2014. The improvement of diagnosis and detection capacity may influence the number of reported SFTS cases. Nevertheless, the increase number of SFTS cases inform that more cases will be reported in future which prompt the research of prevention methods for SFTSV infection including the SFTSV vaccine.

Occupation distributions of SFTS cases in different years were similar and the majority of cases were farmers who lived in rural areas. As SFTSV is believed to be transmitted by *Haemaphysalis longicornis* ticks^15^, higher tick density in rural areas and more outdoor activities of farmers may contribute to the result. Farmers are the key population for the prevention of SFTS.

Although most SFTS cases aged between 40 and 80, the age distributions of cases in different years were significantly different and the median age slightly increased. These high prevalence age group are in agreement with corresponding data from other studies^16–18^. Elderly people might have decreased immune function and comorbidities with chronic diseases. If they are infected with SFTSV, they may get severe diseases. It is thereby that more elderly cases were identified. Ding *et al*. also reported that people at all age groups were susceptible to SFTSV, but only old people got severe disease and needed to be hospitalized or even died of SFTSV infection^19^. Besides this factor, demographic features of residents in areas where SFTS cases occurred may also influence the age distribution. Generally, young adults in rural areas go to cities to earn money and then return to their hometown for the spring festival. As a result, the majority of residents of rural areas between the months of March and November are elderly people. Furthermore, high prevalence of SFTSV among elderly people indicate that SFTSV is a new virus and the immunity rate is not very high among them.

SFTSV was firstly identified in Henan and Hubei, then it was identified in Anhui, Shandong, Jiangsu, Zhejiang, and Liaoning. Up to date, the overwhelming majority of SFTS cases were reported in these 7 provinces which all locate in the central part of China. We speculate that the weather in these 7 provinces is more suitable for the survival and breeding of ticks, and the transmission of SFTSV. But further study is needed to study the relation between meteorological factors and SFTSV incidence. Another factor may all influence the geographical distribution. More attentions have been paid, and more researches have been done in these 7 provinces where the capacity of SFTSV detection is developed.

Although the number of affected province in different years were similar, the number of affected counties increased sharply every year and SFTS cases were also reported in other provinces besides its endemic areas. These results highlight that the fact that the geographic range of SFTSV has apparently expanded in China, which is valuable information for consideration in national planning on SFTS control and prevention. More investigation should be conducted to confirm whether SFTS is endemic in the provinces where sporadic cases were reported.

Most SFTS cases occurred from April to October, but the seasonal distributions of different provinces were significantly different. Provinces in south regions showed earlier epidemic peak and longer epidemics durations. These are also related to the climate of these provinces. These results suggest that control measures should be conducted during different periods in different provinces.

To our great disappointment, the interval from illness onset to confirmation from 2011 to 2016 were not shortened. These were consistent with corresponding results of another study^20^. This may be due to SFTS patients’ low incomes and poor access to medical care because most of them were from rural areas. Rural residents commonly visit local hospitals or outpatient clinics in villages or towns. These hospitals do not have the capability to identify and cure SFTSV infection. They have to go to urban areas for hospitals with better facilities for further care. The majority of hospitals don’t have the capacity to detect SFTSV and most samples are transported to municipal centers for disease control and prevention (CDC) or provincial CDC for testing. These factors prolong the interval from illness onset to confirmation. However, the interval from illness onset to confirmation is very important for the cure of SFTS cases. Sun *et al*. reported that an increase in interval from illness onset to confirmation by 3 days was associated with fatality with an OR of 1.996^21^. These inform that detection capability of SFTSV should be established not only in centers for diseases control and prevention but also in different hospitals to decrease the case fatality rate.

The fatality rate decreased from 2011 to 2016, but it remained very high in 2016. In the analysis of factors associated with SFTS fatal outcome, fatality rates of cases in different years and different provinces were significantly different. Provinces with higher number of SFTS cases showed lower fatality rate. For example, Henan presented the highest number of SFTS cases and the lowest fatality rate. One reason is that physicians in these provinces are more experienced to cure SFTS, so cases are more probable to be cured in these provinces. Another reason is that the detection capability is developed in these provinces. Not only severe cases but also mild cases are identified in these provinces. As a result, more cases were identified and fatality rates were low in these provinces. The high fatality rate suggest that it is urgent to study effective treatments for SFTS.

Previously studies have reported that case fatality rate of SFTS increases with age^10, 22–24^. Our study also indicated that age was related to the fatality rate. Some factors associated with age including decreased immune function and comorbidities with chronic diseases may be relative to SFTS fatal outcome. Nevertheless, our findings further suggest that more intense treatment should be administered to elderly SFTS cases when they were hospitalized.

According to our results, we suggest that some measures should be done to prevent and control SFTS. First, health education should be enhanced. People should avoid go to hilly areas where ticks exist in epidemic seasons. If they have to go to these areas, they should dress light color clothes and check whether ticks bite them when they leave these areas. Second, hospitals in SFTS endemic areas should improve capacity of SFTSV detection. This is helpful for the early diagnosis and the decrease of fatality of SFTS patients. Finally, some measures should be conducted to decrease tick density in endemic areas during epidemic seasons.

There are several limitations in our study. First, the data used were collected from China Information System for Diseases Control and Prevention which is passive surveillance. Some factors including detection capability, reporting methods, availability of health facilities may influence the data quality. Second, there are too many floating population in some provinces of China. They go to urban areas and come back hometowns irregularly. So we can’t get the real number of people who lived in rural areas during high risk seasons which is disadvantageous to analyze incidence of SFTS among people with different demographic characteristic.

To the best of our knowledge, this is the first report on the changing epidemiological characteristics of SFTS in China. We found that the number of SFTS cases increased, the affected areas expanded and some demographic characteristics changed year by year indicating that surveillance and control strategies should be adjusted to account for these changes. Unfortunately, surveillance and control strategies are largely inadequate in China. For example, there are no national sentinel vector surveillance project for ticks and no national control program for ticks in China. Therefore, it is essential for China to put more effort into ticks surveillance and control. The increased interval from illness onset to confirmation further suggests that it is urgent to improve the diagnosis capability of physicians for early diagnosis. More researches should be conducted and more attention should be paid to respond the changed characteristics of SFTS.

## References

[1] Yu X (2011). Fever with thrombocytopenia associated with a novel bunyavirus in China. N Engl J Med..

[2] Xu B (2011). Metagenomic analysis of fever, thrombocytopenia and leukopenia syndrome (FTLS) in Henan Province, China: discovery of a new bunyavirus. PLoS Pathog..

[3] Sun J (2014). Epidemiological characteristics of severe fever with thrombocytopenia syndrome in Zhejiang Province, China. Int J Infect Dis..

[4] Liu K (2015). A national assessment of the epidemiology of severe fever with thrombocytopenia syndrome, China. Sci Rep..

[5] Liu Q, He B, Huang SY, Wei F, Zhu X (2014). Severe fever with thrombocytopenia syndrome, an emerging tick-borne zoonosis. Lancet Infect Dis..

[6] Zhang YZ, Zou Y, Fu ZF, Plyusnin A (2010). Hantavirus infections in humans and animals, China. Emerg Infect Dis..

[7] Zhang LJ (2008). Nosocomial transmission of human granulocytic anaplasmosis in China. JAMA..

[8] Denic S (2011). Acute thrombocytopenia, leucopenia, and multiorgan dysfunction: the first case of SFTS bunyavirus outside China?. Case Rep Infect Dis..

[9] McMullan L (2012). A new phlebovirus associated with severe febrile illness in Missouri. N Engl J Med..

[10] Shin J, Kwon D, Youn SK, Park JH (2015). Characteristics and factors associated with death among patients hospitalized for severe fever with thrombocytopenia syndrome, South Korea, 2013. Emerg Infect Dis..

[11] Takahashi T (2014). The first identification and retrospective study of severe fever with thrombocytopenia syndrome in Japan. J Infect Dis..

[12] Ding F (2013). Epidemiologic features of severe fever with thrombocytopenia syndrome in China, 2011-2012. Clin Infect Dis..

[13] The national guidelines for control and prevention of severe fever with thrombocytopenia syndrome. http://www.moh.gov.cn/mohwsyjbgs/s8348/201010 /49272.shtml. Beijing, Chinese Ministry of Health. Accessed 29 September 2010.

[14] Fu Y (2016). Phylogeographic analysis of severe fever with thrombocytopenia syndrome virus from Zhoushan Islands, China: implication for transmission across the ocean. Sci Rep..

[15] Luo LM (2015). Haemaphysalis longicornis Ticks as reservoir and vector of severe fever with thrombocytopenia syndrome virus in China. Emerg Infect Dis..

[16] Yu B (2012). Epidemiological study on data involving 61 hospitalized cases with Huaiyangshan hemorrhagic fever in Wuhan, Hubei province. Chin J Epidemiol..

[17] Liu L (2012). Epidemiologic analysis on severe fever with thrombocytopenia syndrome in Hubei province, 2010. Chin J Epidemiol..

[18] Xu B (2011). Metagenomic Analysis of Fever, Thrombocytopenia and Leukopenia Syndrome (FTLS) in Henan Province, China: Discovery of a New Bunyavirus. PLoS Pathog..

[19] Ding S (2014). Age Is a Critical Risk Factor for Severe Fever with Thrombocytopenia Syndrome. PLoS One..

[20] Li Y, Zhou H, Mu D, Yin W, Yu H (2015). Epidemiological analysis on severe fever with thrombocytopenia syndrome under the national surveillance data from 2011 to 2014, China. Chin J Epidemiol..

[21] Sun J (2016). Factors associated with Severe Fever with Thrombocytopenia Syndrome infection and fatal outcome. Sci Rep..

[22] Guo C (2016). Epidemiological and clinical characteristics of severe fever with thrombocytopenia syndrome (SFTS) in China: an integrated data analysis. Epidemiol Infect..

[23] Liu K (2014). Epidemiologic features and environmental risk factors of severe fever with thrombocytopenia syndrome, Xinyang, China. PLoS Negl Trop Dis..

[24] Zhang Y (2012). Hemorrhagic fever caused by a novel Bunyavirus in China: pathogenesis and correlates of fatal outcome. Clin Infect Dis..

